# Human Infection of Methicillin-Susceptible *Staphylococcus aureus* CC398: A Review

**DOI:** 10.3390/microorganisms8111737

**Published:** 2020-11-05

**Authors:** Kevin Bouiller, Xavier Bertrand, Didier Hocquet, Catherine Chirouze

**Affiliations:** 1 Maladies Infectieuses et Tropicales—Centre Hospitalier Universitaire, 25030 Besançon, France; cchirouze@chu-besancon.fr; 2 UMR-CNRS 6249 Chrono-Environnement, Université Bourgogne Franche-Comté, 25000 Besançon, France; bertrand@chu-besancon.fr (X.B.); dhocquet@chu-besancon.fr (D.H.); 3 Hygiène Hospitalière—Centre Hospitalier Universitaire, 25030 Besançon, France

**Keywords:** ST398, methicillin-susceptible *Staphylococcus aureus*, CC398, blood stream infection

## Abstract

*Staphylococcus aureus* (SA) belonging to the clonal complex 398 (CC398) took a special place within the species due to its spread throughout the world. SA CC398 is broadly separated in two subpopulations: livestock-associated methicillin-resistant SA (MRSA) and human-associated methicillin-susceptible SA (MSSA). Here, we reviewed the global epidemiology of SA CC398 in human clinical infections and focused on MSSA CC398. The last common ancestor of SA CC398 was probably a human-adapted prophage φSa3-positive MSSA CC398 strain, but the multiple transmissions between human and animal made its evolution complex. MSSA and MRSA CC398 had different geographical evolutions. Although MSSA was present in several countries all over the world, it was mainly reported in China and in France with a prevalence about 20%. MSSA CC398 was frequently implicated in severe infections such as bloodstream infections, endocarditis, and bone joint infections whereas MRSA CC398 was mainly reported in skin and soft tissue. The spread of the MSSA CC398 clone is worldwide but with a heterogeneous prevalence. The prophage φSa3 played a crucial role in the adaptation to the human niche and in the virulence of MSSA CC398. However, the biological features that allowed the recent spread of this lineage are still far from being fully understood.

## 1. Introduction

*Staphylococcus aureus* (SA) is a leading cause of morbidity worldwide [1]. During the past 20 years, livestock animals, especially pigs, have been identified as a reservoir of methicillin-resistant SA (MRSA) isolates that clustered in clonal complex 398 (CC398). MRSA CC398 has emerged worldwide and has been found to colonize and infect animals and humans [2]. Recent genomic analyses have demonstrated the existence of two main subpopulations within the CC398: an ancestral human-adapted clade with the integrase group 3 prophage (φSa3) containing the immune evasion cluster (IEC) genes and the erythromycin-resistant gene erm(T) and an animal-associated clade associated with the absence of φSa3, and the acquisition of resistance to tetracycline and methicillin [1,3,4]. Severe infections, such as bloodstream infection (BSI), endocarditis, osteomyelitis and necrotizing pneumonia or mild infections such as skin and soft-tissue infections (SSTI) were reported in both populations of CC398. However, the majority of publications examining SA CC398 to date have focused on livestock-associated MRSA.

In this review, we first discussed the evolution of SA CC398 and its epidemiology in clinical infections in human and then focused on methicillin-susceptible SA (MSSA) CC398.

## 2. Origin and Evolution of the Different Lineages of CC398

Although a history of exposure to livestock has been identified as one of the main risk factors for human infections with CC398, an increasing number of cases are emerging in patients with CC398 infection with no such history [5,6,7]. Epidemiological data suggested a possible transmission of this strain between humans, which could explain its spread [6,8,9].

Microarray studies of CC398 have shown a distinction between livestock-associated infections and those associated with human-to-human transmission in terms of their distribution of mobile genetic elements (MGEs) and antibiotic resistance [3,9].

SA CC398 was then separated into two subpopulations: a human (Hu) clade and a livestock-associated (LA) clade. The two clades are characterized by different prophages. LA-MRSA CC398 isolates commonly carry phages φ2, φ6, or φAvb [1,10,11]. By contrast, isolates belonging to the human clade contain the φSa3 prophage carried the human-specific immune evasion cluster (IEC) in particular genes chp (chemotaxis inhibitory protein; CHIPS) and scn (staphylococcal complement inhibitor; SCIN) [1,9]. Additional prophages elements are believed to distinguish isolates of the ancestral subpopulation from the emerging human subpopulation, such as φMR11-like prophage [12,13]. Moreover, LA-CC398 had tetracycline resistance with the presence of the *tet*(M) gene, and methicillin resistance with the presence of SCC*mec*, while Hu-MSSA CC398 had erythromycin resistance with *erm*(T) gene.

The advent of whole-genome sequencing (WGS) has allowed the reconstruction of the evolution of this clone. WGS analyses revealed a possible human origin for LA-CC398, followed by the emergence of methicillin and tetracycline resistance driven by antibiotic pressure in animal [1]. Indeed, according to Price et al. [1], the last common ancestor of SA CC398 was probably a human-adapted IEC-positive MSSA CC398 strain, which at a later stage acquired SCC*mec*, leading to the emergence of human MRSA CC398 strains. At some point, ancestral MSSA CC398 strain jumped to livestock, which was accompanied by the loss of IEC and the acquisition of *tet*(M), conferring resistance to tetracycline and later on by the acquisition of SCC*mec*, conferring resistance to methicillin, leading to the emergence of livestock-associated MRSA CC398. However, a more recent quantitative time-scaled phylogeny, indicated that both Hu and LA-SA CC398 emerged in parallel around 1970 [14]. Ward et al. [14] supported the existence of distinct human- and livestock-associated clades that emerged at similar times, with some interspecies transmission in both directions. The combined MLST sequence from a number of sequence types (STs) confirmed that SA may jump between human and livestock hosts in both directions [15]. In the same way, the global CC398 phylogeny presented by Chen et al. suggested the presence of some human CC398 isolates in the animal source clade and vice versa, which further supports the spread of CC398 between animals and humans [16].

Finally, in view of the frequent transfers of CC398 between humans and animals, with some differences according to the country, a detailed analysis of the dissemination of CC398 between individual countries with a same sampling methodology should be done, to better understand the evolution and the spread of this clone.

**Figure 1:**
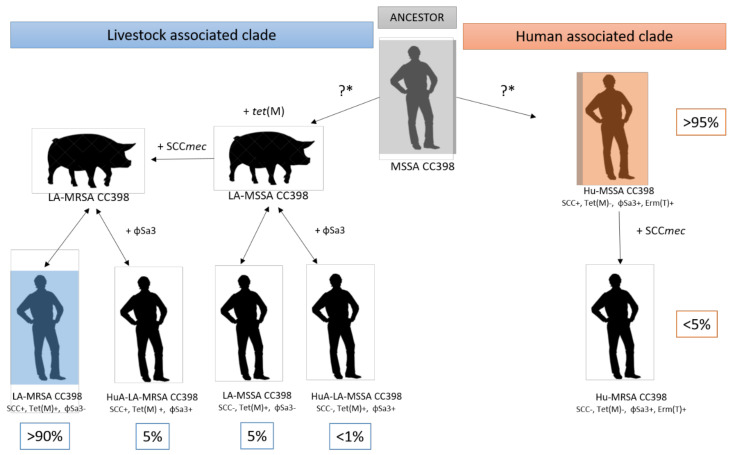
Schematic representation of the evolution of different subpopulations within the CC398 lineage. The last common ancestor of *Staphylococcus aureus* (SA) CC398 was probably a human-adapted immune evasion cluster (IEC)-positive methicillin-susceptible SA (MSSA) CC398 strain, which at a later stage acquired SCC*mec*, leading to the emergence of human methicillin-resistant SA (MRSA) CC398 strains. At some point, the ancestral MSSA CC398 strain jumped to livestock, which was accompanied by the loss of IEC and the acquisition of tet(M), conferring resistance to tetracycline and later on by the acquisition of SCC*mec*, conferring resistance to methicillin, leading to the emergence of livestock-associated MRSA CC398. However, Ward et al. [14] supported the existence of distinct human- and livestock-associated clades that emerged at similar times, which was represented by “?*” in the figure. Because some interspecies transmission in both directions was described, a double-arrow was used to represent the different livestock-associated clade subpopulations. These transmissions were probably responsible for the acquisition of the prophage φSa3 in the LA clade, which differentiated human (Hu) SA CC398 subpopulations from the adapted Humans (HuA) livestock-associated (LA) SA CC398 subpopulations.

We proposed a model of evolution of SA CC398, into the two different subpopulations: animal and human clade (Figure 1). The animal clade was separated into MRSA and MSSA LA CC398. Human-adapted LA CC398 was defined according to the acquisition of the φSa3 prophage.

## 3. Epidemiology of SA CC398 Infections

**Figure 2:**
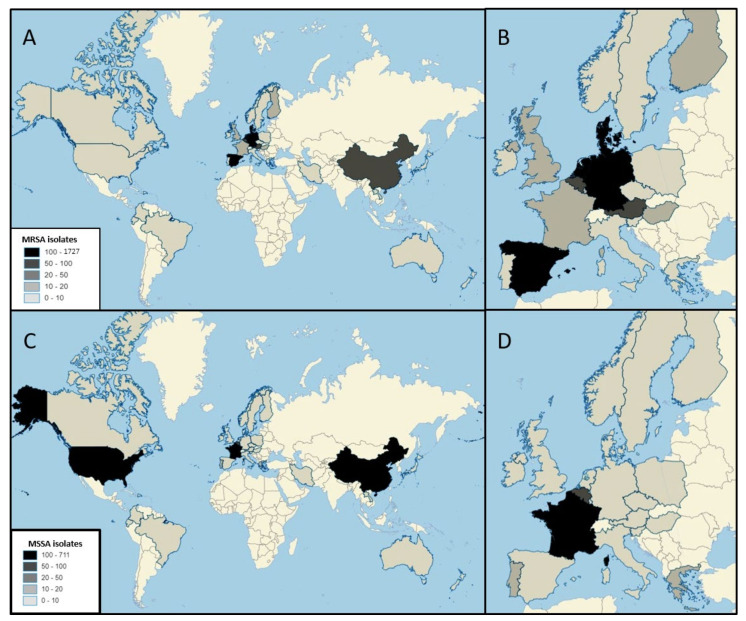
Number of MRSA (**A**,**B**) and MSSA (**C**,**D**) CC398 isolates from clinical infection in the world and in Europe. MRSA: Denmark (*n* = 1727), Germany (*n* = 600), The Netherlands (*n* = 446), Spain (*n* = 310), China (*n* = 78), Belgium (*n* = 77), Austria (*n* = 67), Sweden (*n* = 2), Slovenia (*n* = 6), France (*n* = 18), Luxembourg (*n* = 16), United Kingdom (*n* = 14), Hungary (*n* = 12), Finland (*n* = 10), New Zealand (*n* = 7), Italy (*n* = 6), Greece (*n* = 4), Canada (*n* = 4), United States (*n* = 3), Japan (*n* = 2), Hong Kong (*n* = 2) Laos (*n* = 1), Japan (*n* = 1), Australia (*n* = 1)**,** Norway (*n* = 1), Ireland (*n* = 1), Poland (*n* = 1); MSSA: France (*n* = 711), China (*n* = 255), United States (*n* = 117), Belgium (*n* = 58), Portugal (*n* = 18), Greece (*n* = 17), The Netherlands (*n* = 7), Dominican Republic (*n* = 7), Iran (*n* = 6), Germany (*n* = 6), Spain (*n* = 4), Denmark (*n* = 4), Laos (*n* = 4), Ireland (*n* = 2), Colombia (*n* = 2), Italy (*n* = 1), Ecuador (*n* = 1), Brazil (*n* = 1).

The limited molecular epidemiology of large collections of MSSA has probably precluded an accurate assessment of the global spread of CC398 [9]. Since SA CC398 was first reported from the colonization of pigs and of farmers in France in the late nineties [2], human SA CC398 infections have been sporadically reported all over the world (Figure 2), whereas the majority of them have been reported in Europe (mostly in France) and China [17,18,19,20,21,22,23].

A variety of MRSA CC398 infections has been documented, ranging from relatively minor or localized infections (SSTI) to more serious or invasive infections (including bloodstream infection (BSI), osteomyelitis, pneumonitis, and endocarditis) [24]. Despite the diverse array of infection types, it has been suggested that MRSA CC398 may not cause as much severe diseases as MSSA CC398 [25]. Details on MSSA CC398 studies were presented in the Table 1.

The analysis of the publications that have specified the clinical diagnosis of SA CC398 infections is summarized in Table 2. BSI, endocarditis, and bone joint infections (BJI) were reported more frequently in MSSA CC398 studies, whereas SSTI, respiratory tract and urinary tract infections were reported more frequently in MRSA CC398 studies. However, most countries do not perform MSSA surveillance, and when they do, they focus on invasive isolates, meaning that MSSA SSTIs are likely to be underreported in the literature.

## 4. Frequency and Geographic Distribution of MSSA CC398 Infections

### 4.1. In France

In France, since the first description of MSSA CC398 by Armand-Lefevre et al., 16 publications with data on MSSA CC398-related infections have been published, and the proportion of CC398 among total MSSA infections ranged from 1.9 to 38.3% depending on the date of the study and type of infections [7,12,17,18,19,20,29,30,31,32,33,34,35,36,37,78].

A population-based survey of patients with MSSA infective endocarditis (2008) and MSSA BSI (2006–2007) revealed that 7 of 170 (4.1%) MSSA isolates belonged to CC398 [34]. Valentin-Domelier et al. reported 17 MSSA CC398 BSIs during a four year survey in three non-contiguous French regions with increased incidence by seven-fold (0.002 per 1000 patient days in 2007 vs. 0.014 in 2010) [7]. Bonnet et al. found that ST398 was the most frequent clone (8.8%) in a collection of 182 BSI-related MSSA isolates in 2013 [20]. In a French University hospital, the proportion of CC398 among BSI-related MSSA isolates has steadily increased in this past decade: from 4.6% in 2010 to 13.8% in 2014, and 20.2% in 2017 [17,18].

Concerning BJI, a retrospective study in 2008 found that only 1.9% of MSSA were ST398 [31]. Valour et al. found that 68 of the 485 MSSA prosthetic joint infection isolates (14%) belonged to CC398 in a multicenter study in France between 2009 and 2012 [35], with a geographical heterogeneity and a regional prevalence ranging from 3.1 to 23.5%.

In patients with diabetic foot infection (DFI), MSSA ST398 represented 26 of 166 (15.7%) and was the main lineage isolated. Moreover, it was identified exclusively in cases with osteomyelitis [32]. These results were confirmed by a multicenter study of patients with DFI, with a prevalence of 21.7% (35/161) that reached 38.3% in patients with diabetic foot osteomyelitis only [33]. Recently, our group reported an increasing trend of prevalence of MSSA CC398 in BJI over a 8 year period (from 4% in 2010 to 26% in 2017), echoing what we observed in BSIs [30]. Overall, since the incidence of MSSA CC398 steadily increased in the last decade, it became a predominant *SA* lineage responsible for infections in France.

### 4.2. In Europe

In Europe, MSSA CC398 represented 2.1% of the 565 MSSA isolates with data of spa typing from invasive infections (mainly BSI) collected in 26 countries during the period 2006–2007 [25]. These isolates were retrieved in eight different countries which suggested the spread of this clone in Europe. However, in a retrospective study from Germany, the prevalence of MSSA CC398 isolates from 2006 to 2012 remained low (0.14% among isolates from infection in humans) [38]. As in a large collection of 610 consecutive isolates of *SA* BSI episodes in a multicenter study in The Netherlands that only retrieved two MSSA CC398 (0.3%) isolates during the period 2010–2011 [47]. Rijnders et al. also found a limited prevalence of CC398 among *SA* isolates in intensive care units (2 of 936, 0.2%) in this country [46]. In Belgium, among 212 MSSA isolates collected during a national survey in 2008, five belonged to CC398 and were retrieved from patients located in different regions [27]. In southern Europe, in a collection of 465 MSSA isolates from infection and colonization collected in Portugal over a 19 year period (1992–2011), 14 (3%) late isolates found during the period 2010–2011, were MSSA ST398-t571, indicating the recent emergence of this lineage in this country [49]. In Greece, the first case of an MSSA CC398 infection was described in 2011 in a BSI [40]. Then, Sarrou et al. reported, among a large collection of 492 clinical MSSA isolates from infected humans during the period 2012–2013, 2.6% of MSSA CC398 (13/492 isolates) [42].

### 4.3. In China

As observed in France, MSSA CC398 is the most prevalent *SA* lineage in China with a prevalence varying from 5.5 to 26.6% [21,22,23,53,54,55,56,57,58,59,60,61,62,63,64]. This lineage was equally retrieved in patients with SSTI or BSI. The prevalence of MSSA CC398 increased from 8.9 to 26.6% in 5 years in the Hainan region, among MSSA isolates from BSI [22]. These results were confirmed in a multicentric study in 12 provinces, distributed in distant geographic areas with a prevalence of 7.8% in 2010 and 10.1% in 2016, among bacteremia isolates [60]. In patients with SSTI, the prevalence of MSSA CC398 increased from 11.6 (11/95) to 17.4% (8/46) between 2011 and 2015 [57,58]. The first isolate was reported during the period 1994–1998 [64].

### 4.4. In America

MSSA CC398 infections are also observed in America. In South America, MSSA ST398 *spa* type t571 has been described in Colombia as causing infection in a woman without livestock association and in Ecuador from a patient with BSI [69,72]. From 70 consecutively BSI and non-consecutive isolates from different clinical infections, only one (1.4%) isolate from BSI was MSSA CC398 [72]. Recently, Jewel et al. reported a case of severe disseminated infection caused by MSSA CC398 in an immunocompetent man, shortly arrived in the United Kingdom from Colombia [70]. Another case was reported in an oncology patient in Sao Paulo, Brazil, with the first Cfr-producing MSSA ST398 from a patient with a fatal pneumonia [68]. In Dominican Republic and Martinique, CC398 prevalence reached, respectively, 7.8% (7/90) and 10.4% (9/87) among clinical MSSA [71].

In the United States, most cases of MSSA CC398 infections are reported in New York City [75,76,77]. In Northern Manhattan, the rate of MSSA CC398 in non-invasive infections was 4% (64/1607), and 2.5% (4/160) in BSI [8,9]. During a 7 year period (2004–2010), Mediavila et al. reported 13/4167 (0.3%) MSSA CC398 clinical isolates, in the New York City area [75].

Finally, MSSA CC398 was retrieved all over the world but with different prevalence depending on the country. France and China were countries where this clone has been most studied and where it is most frequently isolated from human infections. However, most countries do not perform MSSA surveillance, and the disease burden of MSSA CC398 at the national and international level is therefore largely unknown.

## 5. Types of MSSA CC398 Infections

### 5.1. BSI

In France and China, respectively, the two most affected countries, MSSA CC398 is responsible for BSI, ranging from 4.1 to 13.4%, and from 5.4 to 14.9% of all BSI [7,17,18,20,23,34,52,55,59,60,61]. Even though MSSA CC398 infective endocarditis (IE) has been described, CC398 was equally prevalent between IE and non-IE bacteremia and represented 5.6% of MSSA endocarditis isolates [34]. Among 105 MSSA CC398 infections, endocarditis was diagnosed in seven (8.4%) cases [19].

### 5.2. BJI

BJI related to CC398 included principally prosthetic joint infection (PJI) and diabetic foot osteomyelitis (DFO) [33]. MSSA CC398 was the dominant clone in patients with DFO, accounting for 17.9% among all SA BJI and 38% among all SA DFO [30,33]. The frequency of CC398 SA PJI isolates varied from 1.8 to 18.1% of all SA PJI [30,31,35]. Moreover, Senneville et al. showed that *SA* CC398 isolates were significantly more frequent in osteomyelitis than in SSTI patients with diabetic foot infection, suggesting a particular tropism of this clone for bone [33]. However, CC398 had a low potential for osteoblast invasion in ex vivo models, suggesting unidentified virulence factors [35].

### 5.3. Respiratory Tract Infections

MRSA CC398 seems to be significant in respiratory tract infections (RTI) [24]. It could be related to the mode of transmission, with close contact with animals. In contrast, fewer cases of MSSA CC398 RTI have been reported [7,8,16,19,20,27,29,37,39,50,55,61,62,68,74]. Hence, a French study of 89 clinical isolates of MSSA CC398 reported that 12% were isolated from pneumonia, 22% from BSI and 29% from SSTI [19]. However, this collection included isolates voluntarily sent by French microbiology laboratories for further characterization because of a particular antibiotic-resistant phenotype or unusual infections, or in the setting of targeted studies. Thus, the collection does not represent the entire MSSA CC398 population in France. Similarly, pneumonia represented 14% of all MSSA CC398 infections (excepted BSI) in New York [8], and MSSA CC398 represented 4.9% of all SA respiratory tract infections in China [61]. Moreover, Song et al. reported that 10.3% of *SA* pneumonia in pediatric inpatients was due to MSSA CC398 [62]. RTI was also reported as the portal of entry of BSI in several studies in which ≤ 1/4 of the MSSA CC398 BSI isolates were of pulmonary origin [7,20,50]. Two fatal cases of pneumonia have been reported in France and Brazil [29,68].

### 5.4. SSTI

SSTI seems to be the predominant infection linked to MRSA CC398. However, this over-representation could be biased due to the specific surveillance of MRSA infections in several countries, which lacks for MSSA. Indeed, in a French study of 105 clinical isolates of MSSA CC398, SSTI was the most represented infection (35% of cases), followed by BSI with 26.5% of cases [19]. However, all specimens in this study were voluntarily sent to French National Center for Staphylococci, because of a particular antibiotic-resistant phenotype or unusual infections, which make it impossible to generalize these results. For patients with BSI, a SSTI was the suspected portal of entry in 6.3–11% of the cases, in line with other clonal complexes (CCs) [7,17,20]. The proportions of CC398 among MSSA and MRSA isolated from SSTIs in China were similar (12–17%) [58]. Moreover, MSSA CC398 was present in 1/26 (3.8%) SA isolates colonizing the skin of children with atopic dermatitis [79]. Finally, the predominance of MRSA CC398 in SSTI could be due to a publication bias. However, we suggest that the mode of transmission, with close contact with animals, plays a role in the clinical type of MRSA infection.

### 5.5. Community-Acquired or Healthcare-Associated Infection (HAI)

In the study of Bonnet et al., MSSA CC398 BSI were associated with an intravascular device in 56.3% of the cases vs. 31.6% in non-CC398 MSSA BSI [20]. Similarly, Valentin-Domelier et al. showed that BSI related to surgical site infection (SSI) or an intravascular device were found preponderant among CC398 BSI cases (14/18, 78%) [7]. In a retrospective study, MSSA CC398 BSI were more frequent in patients with SSI (22.4 vs. 11.2%) and another study found that patients with MSSA CC398 infections were more likely hospitalized in the past 6 months, suggesting a nosocomial transmission (41 vs. 22%, *p* < 0.01) [8,17]. Moreover, we found that HAI was associated with CC398 MSSA BSI in multivariate analysis, compared to MSSA BSI with other CCs [17]. These data suggested that this clone was possibly related to HAI. However, existing literature found evidence for MSSA CC398 as both a community- and hospital-associated pathogen [8,9,18,37]. Moreover, MSSA CC398 has been described as colonizer of healthy humans, in addition to causing infections [80]. Prevalence studies should be carried out to test whether patients in a hospital setting are more colonized than a healthy population for MSSA CC398.

## 6. Comorbidities

Like most *SA* infections, many case reports of patients with MSSA CC398 described patients with predisposing conditions (e.g., diabetes, advanced age, immunosuppressive treatment). However, some studies showed different characteristics from patients with MSSA CC398 and non-CC398. Indeed, it has been reported that BSIs due to MSSA CC398 more frequently affected fragile patients with a history of neurological disease [17]. Similarly, Uhlemann et al. found that a higher proportion of alcohol abuse and cirrhosis were associated with MSSA CC398 infections and suggested a poorer health status of patients [8]. In the same way, patients with PJI and DFO due to MSSA CC398 had more comorbidities than those infected by MSSA non-CC398 [30]. In the study of Bonnet et al., patients with BSI due to MSSA CC398 more often had diabetes mellitus (31.3 vs. 25%) and immunosuppression (31.3 vs. 28.3%), but the small size of the patient cohort did not allow to reach the significance threshold [20].

## 7. Outcome

As mentioned above, MSSA CC398 causes a wide range of infections, including BSIs. Many studies have reported MSSA CC398 deaths in France. A lethal necrotizing pneumonia caused by Panton–Valentine leukocidin (PVL) producing MSSA CC398 was described in a previously healthy 14 year-old girl [29]. Another patient with a cancer died from a pneumonia with BSI in Brazil without further details of his infection [68]. Two additional deaths were reported, in a 59 year-old male and an 80 year-old male, among 17 patients which had a BSI with MSSA CC398 (11.8%) [7]. Moreover, in a monocentric retrospective study, BSIs due to MSSA CC398 were associated with a high risk of 30 days-mortality than other CC in a multivariate analysis (41.8 vs. 27.6% OR = 2.44) [17]. Ulhemann et al. described a trend to more frequent episodes of secondary invasive disease with CC398 but no difference in the proportion of a fatal outcome (6.3 vs. 4.2% *p* = 0.5) [8]. However, in this study, BSIs were excluded. Conversely, MSSA CC398 PJIs were significantly associated with a lower biological inflammatory syndrome (*p* = 0.035) and lower treatment failure rates (0 vs. 37.3%, *p* = 0.032) than other CCs [35]. These conflicting results may indicate yet unidentified virulence features of MSSA CC398. Alternatively, it may also reflect the underlying comorbidities or an immunosuppressed state of the affected host more than the bacterial virulence itself, as suggested above.

## 8. Antibiotic Resistance

Whereas MRSA CC398 were frequently multiresistant to antibiotic, including tetracycline, secondary to the wide use of this antibiotic in the pig industry, MSSA CC398 was more often only reported as resistant to erythromycin [24].

Uhlemann et al. reported that MSSA CC398 isolates retrieved in humans in New York (USA) were almost always resistant to erythromycin (97%) and clindamycin (97%) [8]. This resistance was very mainly associated with the presence of *ermT* gene [19]. Interestingly, *ermT* was absent in MRSA CC398 from animal origin [81].

Tetracycline resistance was rarely described in MSSA isolates but was frequent in MRSA CC398 due to the presence of *tet*(M). Indeed, only four MSSA CC398 studies showed tetracycline resistance with the presence of *tet*(M) gene [28,31,40,42]. However, the absence of WGS of these isolates did not distinguish LA and Hu clades.

## 9. Virulence Factors

*SA* CC398 were initially distinguished by their peculiar resistance to digestion by *Sma*I, the restriction enzyme most frequently used for PFGE typing [82]. Investigations into the molecular background of MSSA CC398 showed that, most well known staphylococcal virulence genes such as enterotoxins, toxic-shock syndrome toxin or leukocidins are most of the time, lacking [1,35]. However, the 30 day all-cause mortality was higher for patients with CC398 MSSA BSI than for a control group with non-CC398 MSSA BSI [17]. This lineage may represent a more virulent CC398 subtype, as suggested by its higher prevalence in BSI. Genome analysis of CC398 isolates showed that the mobile genetic elements (MGEs) were specific for each population and enabled the differentiation of strains responsible for asymptomatic colonization to invasive infections [12]. Most of the bacterial factors involved in infection severity are MGEs that belong to the accessory genome. The prophage φSa3 contains the immune evasion gene cluster (IEC, which facilitates the escape from human immune response) genes *sak* (coding for a staphylokinase), *chp* (coding for a chemotaxis inhibitory protein; CHIPS), *scn* (coding for a complement inhibitor; SCIN), sea (coding for enterotoxin; SEA) and sep (coding for enterotoxin; SEP). However, only a small proportion of the MRSA CC398 isolates retrieved from infected patients contains the φSa3-associated IEC [1,7,26,42,66,83,84,85,86,87]. This suggests that although IEC is not a prerequisite for infections in humans, it plays a crucial role in the adaptation to the human niche. Moreover, the presence as well as the structure of φSa3 seems to be directly associated with virulence, even though the different genetic backgrounds of the φSa3-positive and φSa3-negative isolates was not take into account in this study [88]. Interestingly, human *SA* CC398 had a higher capacity for binding to human cytokeratin-10 than LA SA CC398, mediated by the clumping factor B (ClfB) and very likely important for nasal colonization of humans [9]. Recently, Laumay and colleagues showed that the introduction of bacteriophages from the genomes of human-adapted *SA* to that of a naive *SA* animal colonizer increased the transcription of *clfA* (encoding the clumping factor A precursor) and *fnbA* (encoding the fibronectin binding protein), which in turn increased the bacterial virulence in a rat model of infectious endocarditis [89]. This study confirmed the role of MGE in the virulence of *SA* CC398.

The PVL-encoding gene is usually absent in MSSA CC398 genomes, but the spread of a PVL-producing CC398 subpopulation in China confirmed the capacity of this clone to acquire virulence genes [21,22,23,55,56,57,59,61,62,63]. Elsewhere, rare cases of PVL-producing MSSA CC398 isolates have been reported in other countries. Hence, severe infections by PVL-positive MSSA CC398 have been reported in New York, Japan and Europe, including the case of the young girl with fatal necrotizing pneumonia [1,7,8,19,26,29,36,64,90]. The proportion of MSSA CC398 isolates that produce PVL ranged from 0 to 13.8% in Europe and the USA, but reached 86.7% in China [21,22,55,56,59,61,62,63,64,66]. The acquisition of such a virulence factor and/or the spread of such isolates are a source of concern in view of the high fitness of MSSA CC398 for humans.

Other virulence factors have been described in CC398 isolates. The presence of genes encoding hemolysins and intracellular adhesion proteins, cap5, together with three genes encoding MSCRAMM (*bbp*, *clfA*, *clfB*) were reported in the genomes of isolates responsible for DFI [33]. Moreover, Liu et al. found that CC398 isolates harbored exfoliatin genes (eta, etb) more frequently than other CC isolates [21]. Enterotoxin and hemolysins genes were also described in two Chinese studies [21,22].

## 10. Conclusions

The spread of the MSSA CC398 ancestral clone, and/or the transmission of a human-adapted clone from animals, played a role in the emergence of *SA* CC398. Prophages have an important role in bacterial virulence and evolution by carrying genes coding for novel characteristics for their host. However, unanswered questions remain on the epidemiology and clinical relevance of the MSSA CC398 sublineage. Moreover, the biological features that allow the recent spread of this lineage in clinical settings of some countries (e.g., China, France) are still far from being fully understood.

## Figures and Tables

**Table 1:** Frequency and clinical diagnosis of MSSA CC398 infections.

Author (Ref No.)	Country/Study Design	Study Period	Selection Criteria	Number of MSSA CC398/All MSSA Tested (%)	Clinical Diagnosis (*n*)
**EUROPE**					
Grundmann et al. [25]	28 European countries	2006–2007	Five successive MSSA	12/565 (2.1)	-
Argudin et al. [26]	Belgian reference laboratory	2013–2014	All isolates sent to the national reference center	51/124 (41.1)	SSTI (27), deep fluids (7) BSI (4), screening (13)
Vandendriessche et al. [27]	Belgium reference laboratory	2008	MRSA and MSSA isolates during a national survey	5/212 (2.4)	RTI (1), BSI (1)
De vries et al. [28]	Denmark reference laboratory	1957–2002	TET-resistant SA isolates from blood	4/17 (23.5)	BSI (4)
Price et al. [1]	Denmark, France The Netherlands, Canada, USA, China	1999–2010	Collection of CC398 isolates	18/18	Infection, only 16 isolates
Rasigade et al. [29]	France	2009	Case report	1/1	RTI (1)
Bouiller et al. [30]	France monocentric	2010–2017	SA BJI	120/821 (14.6)	BJI (120)
Diene et al. [12]	France multicentric	2007–2015	Selection of ST398-BSI among an annual 3 months survey of SA BSI	75/75	BSI (75)
Aubin et al. [31]	France monocentric	2007–2010	SA monomicrobial PJI	1/56 (1.8)	PJI (1)
Sauget et al. [18]	France monocentric	2010–2017	SA isolated from BSIs	162/1209 (13.4)	BSI (162)
Dunyach et al. [32]	France multicentric	2010–2012	Diabetic foot infection	13/58 (22.4)	DFI (13)
Senneville et al. [33]	France multicentric	2008–2010	Diabetic foot infection	35/136 (25.7)	DFI (31), SSTI (4)
Tristan et al. [34]	France multicentric	2008	Collection of SA IE isolates	7/170 (4.1) IE = 5/89 (5.6) non-IE 2/81 (2.5)	IE (5), BSI only (2)
Valenti-Domelier et al. [7]	France multicentric	2007–2010	SA strains from BSI case	17/615 (2.8)	BSI (17)
Valour et al. [35]	France multicentric	2009–2012	SA CC398 prosthetic joint infection	68/485 (14)	PJI (68)
Van der Mee-Marquet et al. [13]	France multicentric	2009	Annual surveys of bloodstream infection	4/4	BSI (4)
Chroboczek et al. [19]	France reference laboratory	1999–2011	CC398 MSSA human isolates	105/105	SSTI (29), BSI (22), RTI (12), BJI (6) SSI (3), other (33)
Rasigade et al. [36]	France reference laboratory	1981–2007	PVL positive MSSA	1/211 (0.5)	-
Brunel et al. [37]	France single center	2011	SA isolates from clinical samples	2/89 (2.3)	RTI (2)
Bonnet et al. [20]	France, monocentric	2013	Patients hospitalized with SA BSI	16/182 (8.8)	BSI *n* = 16
Bouiller et al. [17]	France, monocentric	2010–2014	All SA BSI	67/670 (10)	BSI (67)
Cuny et al. [38]	Germany reference laboratory	2006–2012	SA isolates	4/2890 (0.1) BSI 2/433 (0.5)	BSI (2), SSTI (2)
Busche et al. [39]	Germany; reference Laboratory	-	SA CC398	2/6	RTI (1), SSTI (1),
Drougka et al. [40]	Greece	2011	Case report	1/1	Catheter-related BSI (1)
Sarrou et al. [41]	Greece monocentric	2012–2017	Consecutive SA isolates from clinical samples	1/81 (1.2)	-
Sarrou et al. [42]	Greece multicentric	2012–2013	CC398 S. aureus clinical isolates	15/511 (2.9)	SSTI (10), BSI (5)
Brennan et al. [43]	Ireland reference laboratory	2010–2014	SA CC398	2/2	BSI (2)
Manara et al. [44]	Italy monocentric	2013–2015	SA Clinical isolates in children	1/58 (2)	SSTI (1)
Chlebowicz et al. [45]	The Netherlands	NA	Infection strain not specified	1/2	SSTI (1)
Rijnders et al. [46]	The Netherlands multicentric	1996–2006	SA isolates (maximum, 100 isolates per ICU)	2/936 (0.2)	-
Verkade et al. [47]	The Netherlands multicentric	1996–1998 2002–2005 2010–2011	consecutive episodes of BSI	2/610 (0.3)	BSI (2)
Van Belkum et al. [48]	The Netherlands reference laboratory		Human MSSA and MRSA CC398 isolates	3/3	BSI (3)
Tavares et al. [49]	Portugal multicentric	1992–1993, 1996–1997, 2001, 2009–2010, 2011	Isolates collection of MSSA	18/465 (3.9) colonization and infection	-
Mama et al. [50]	Spain monocentric	2015–2017	S. aureus isolates (first isolate/patient) from blood cultures	4/50 (8)	BSI (4) = IE (1), septic arthritis (1), catheter (1), RTI (1)
**ASIA**					
Huang et al. [51]	China/Taiwan monocentric	1995–2017	Collection data	3(1.2)	ENT (3)
Chen et al. [16]	China multicentric	1999–2016	4 MRSA and 4 MSSA ST398	4/4	BSI (2), RTI (1), SSTI (1)
Gu et al. [52]	China monocentric	2013–2018	Randomly selected (20 isolates each year)	10/67 (14.9)	BSI (10)
He et al. [53]	China monocentric	2005–2014	Clinical SA ST398 isolates randomly selected	8/8	Respiratory and cutaneous
Jiang et al. [54]	China monocentric	2009–2010	CA purulent SSTI	1/12 (8.3)	SSTI (1)
Song et al. [55]	China monocentric	2005–2010	Non-duplicated S. aureus isolates randomly selected	11/166 (6.6)	RTI (1), BSI (6), SSTI (4)
Yao et al. [56]	China monocentric	2002–2008	SA SSTI	1/51 (2)	SSTI (1)
Gu et al. [57]	China multicentric	2011–2013	SA isolates from SSTI	11/95 (11.6)	SSTI (11)
Gu et al. [58]	China multicentric	2014–2015	SA isolates from SSTI	8/46 (17.4)	SSTI (8)
He et al. [59]	China multicentric	2014–2016	SA isolates from blood and wound	7/130 (5.4)	BSI (1), SSTI (6)
He et al. [23]	China multicentric	2010–2011	Consecutive, non-duplicate S. aureus strains from blood	9/124 (7.2)	BSI (9)
Li et al. [60]	China multicentric	2013 2016	Consecutive, non-duplicate SA bacteremia isolates	24/264 (9)	BSI (24)
Li et al. [22]	China multicentric	2013–2014 2018–2019	Consecutive and non-duplicate SA isolates	30/151 (19.9)	-
Liang et al. [61]	China multicentric	2015–2018	Unduplicated SA clinical isolates	10/67 (14.9)	SSTI (7), RTI (2), BSI (1)
Liu et al. [21]	China multicentric	2011–2012	Consecutive and non-duplicated SA isolates	15/107 (14)	-
Song et al. [62]	China multicentric	2014–2015	Pediatric inpatients with pneumonia caused by SA	6/58 (10.3)	RTI (1)
Zhao et al. [63]	China multicentric	2009–2010	Consecutive outpatients with SSTIs	28/159 (17.6)	SSTI (28)
Chen et al. [5,6,7,64]	China single center	1994–2008	Non-repetitive SA isolates	31/164 (18.9)	-
Tayebi et al. [65]	Iran	2019	Collection data	6/85 (7.1)	-
Yeap et al. [66]	Laos monocentric	2012–2014	Random sample of SA SSTI	4/93 (4.3)	SSTI (4)
Chen et al. [67]	Taiwan single center	2015	Clinical SA isolates from children	3/131 (2.3)	-
**AMERICA**					
Gales et al. [68]	Brazil	2015	Case report	1	RTI (1)
Jiménez et al. [69]	Colombia	2009	Case report	1/1	BSI (1)
Jewell et al. [70]	Colombia/England	2019	Case report	1/1	BSI (1)
Uhlemann et al. [71]	Dominican Republic (DR) and Martinique (M)	2007–2008	Clinical S. aureus isolates	Total: 16/177 (9)	-
Zurita et al. [72]	Ecuador multicentric	2005–2007 2010–2013	Consecutively BSI and non-consecutive isolates from different clinical infections	1/70 (1.4)	BSI (1)
Wardyn et al. [73]	USA	2011	Case report	1	SSTI (1)
Nair et al. [74]	USA multicentric	2011–2013	Twenty clinically MRSA/MSSA infection isolates per month	17/601 (3)	SSTI (15), ENT (1) RTI (1)
Mediavilla et al. [75]	USA monocentric	2004–2010		13/4167 (0.3)	BSI (4), SSTI (9)
Orscheln et al. [76]	USA monocentric	1999–2007	All routine MSSA “abscess” or “wound” in children hospital	1/32 (3.1)	SSTI (1)
Uhlemann et al. [9]	USA monocentric	-	MSSA isolates from outpatients, BSI MSSA isolates, MRSA clinical isolates	12/320 (3.75)	SSTI (4), unknown (4), BSI (4)
Uhlemann et al. [8]	USA monocentric	2010–2012	All MSSA specimens (excluded BSI)	64/1607 (4)	SSTI (40), RTI (9), UTI (2) ENT (3), BJI (3), BSI (4)
Varshney et al. [77]	USA multicentric	-	SA isolates from wounds, and blood cultures	1/113 (0.9)	BSI (1)
McCarthy et al. [3]	USA, Belgium, The Netherlands, Denmark	2008/2011/2010	SA isolates from human with and without pig contact	30/30	-
Bhat et al. [6]	USA, Dominican Republic multicentric	2007–2008	Sample of anonymous infection and colonization isolates	6/6	Infection unspecified (2) screening (4)

CA; community acquired, BSI: blood stream infection, SSI: Surgical site infection, SSTI: skin and soft tissue infection, RTI: respiratory tract infection, PJI: prosthetic joint infection, PVL: Panton-Valentine Leukocidin, UTI: urinary tract infection, ENT: ear, nose and throat, IE: infective endocarditis, DFI: diabetic foot infection, BJI: bone joint infection, SA: *Staphylococcus aureus,* ICU: intensive care unit, TET: tetracycline.

**Table 2:** Clinical diagnosis of patients with MSSA and MRSA CC398 infections (only articles with clinical infection details were included).

	MRSA (*n* = 3742)	MSSA (*n* = 1181)	*p*
Bacteremia	111 (3.4)	383 (32.4)	<0.001
Endocarditis	7 (0.2)	13 (1.1)	<0.001
SSTI	1588 (49)	229 (19.4)	<0.001
Respiratory tract	325 (10)	41 (3.5)	<0.001
Ear, eyes, sinus	31 (1)	9 (0.8)	0.97
Bone joint infection	17 (0.5)	260 (22)	<0.001
Urinary tract infection	88 (2.7)	3 (0.3)	<0.001
Surgical site infection (other BJI)	27 (0.8)	9 (0.8)	1
Intravascular device infection	16 (0.5)	17 (1.4)	<0.001
Other/not specified	1032 (31.8)	217 (18.4)	<0.001

SSTI: skin and soft tissue infection, BJI: bone joint infection.
